# Photocatalytic Degradation of Malachite Green by Titanium Dioxide/Covalent Organic Framework Composite: Characterization, Performance and Mechanism

**DOI:** 10.1002/open.202300209

**Published:** 2024-01-05

**Authors:** Dongmei Yao, Xiaoting Xie, Xuling Liang, Sufen Lu, Hongfang Lai

**Affiliations:** ^1^ Guangxi Key Laboratory of Sericulture Ecology and Applied Intelligent Technology Hechi University Hechi 546300 China; ^2^ Guangxi Colleges Universities Key Laboratory of Exploitation and Utilization of Microbial and Botanical Resources Hechi University Hechi 546300 China

**Keywords:** composite, degradation mechanism, malachite green, photocatalysis, TiO_2_/COF

## Abstract

In this paper, a titanium dioxide/covalent organic framework (TiO_2_/COF) composite was prepared and its photocatalytic removal of dye was investigated. Using tetrabutyl titanate as a titanium source, TiO_2_ nanomaterial was prepared by sol‐gel method. In the presence of TiO_2_, TiO_2_/COF core‐shell composite was prepared by solvothermal synthesis using melamine and 1,4‐phthalaldehyde as ligands. The prepared materials are characterized by SEM, TEM, XPS, XRD, TG, FTIR, BET, EPR, PL, and UV‐Vis‐DRS techniques. Using malachite green as a model of dye wastewater, the photocatalytic degradation performance of TiO_2_/COF composites was investigated under the irradiation of ultraviolet light. The results show that the modification of COF significantly improves the photocatalytic efficiency of TiO_2_, the degradation rate increases from 69.77 % to 93.64 %, and the reaction rate constant of the first‐order kinetic equation is increased from 0.0078 min^−1^ to 0.0192 min^−1^. Based on the free radical capture experiment, the photocatalytic degradation mechanism of TiO_2_/COF was discussed, and the feasibility of its photocatalytic degradation of malachite green was theoretically clarified. Accordingly, a simple and practical method for photocatalytic degradation of malachite green was constructed, which has potential application value in the degradation of dye wastewater.

## Graphical abstract

In this paper, a titanium dioxide/covalent organic framework (TiO_2_/COF) composite was prepared and its photocatalytic removal of dye was investigated. Using tetrabutyl titanate as a titanium source, TiO_2_ nanomaterial was prepared by sol‐gel method. In the presence of TiO_2_, TiO_2_/COF core‐shell composite was prepared by solvothermal synthesis using melamine and 1,4‐phthalaldehyde as ligands. The prepared materials are characterized by SEM, TEM, XPS, XRD, TG, FTIR, BET, EPR, PL, and UV‐Vis‐DRS techniques. Using malachite green (MG) as a model of dye wastewater, the photocatalytic degradation performance of TiO_2_/COF composites was investigated under the irradiation of ultraviolet light. The results show that the modification of COF significantly improves the photocatalytic efficiency of TiO_2_, the degradation rate increases from 69.77 % to 93.64 %, and the reaction rate constant of the first‐order kinetic equation is increased from 0.0078 min^−1^ to 0.0192 min^−1^. Based on the free radical capture experiment, the photocatalytic degradation mechanism of TiO_2_/COF was discussed, and the feasibility of its photocatalytic degradation of malachite green was theoretically clarified. Accordingly, a simple and practical method for photocatalytic degradation of malachite green was constructed, which has potential application value in the degradation of dye wastewater.

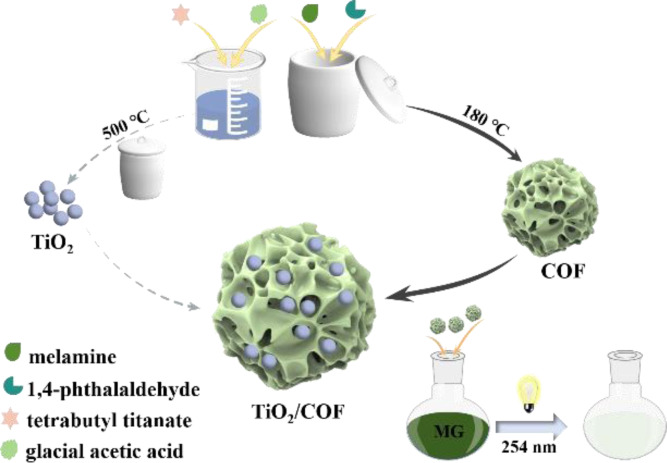

## Introduction

Malachite green (MG) is a green and metallic triphenylmethane dye, which is also one of the hazards of water pollution. Due to the influence of the benzene ring, the molecule has high activity. It can cause cell apoptosis and cancer, and pose a great threat to human life and health.[^1^, ^2^] Therefore, it is very necessary to control the pollution of organic dye wastewater. At present, the removal methods of dye wastewater in water mainly include physical methods (membrane separation technology, adsorption method, etc.), chemical methods (oxidation method, electrochemical method, etc.), and biological methods (aerobic method, anaerobic method, etc.).[^3^, ^4^] These methods have good effects, but there are also some shortcomings, such as slow adsorption rate, high cost, and high requirements for equipment. Therefore, it is of great significance to explore more effective technologies for the advanced treatment of dye wastewater. Photocatalysis technology can use light waves to activate the catalyst to destroy the structure of pollutants.[^5^, ^6^] In recent years, it has attracted the attention of many researchers because of its advantages such as no secondary pollution, no toxicity, and recycling.^[7]^ Among them, TiO_2_ nanomaterials have the advantages of low cost, low toxicity, and good photocatalytic effect, and become one of the most promising catalysts at home and abroad.[^8^, ^9^] Miao et al^[10]^ synthesized TiO_2_ by a facile hydrothermal method and carried out photocatalytic experiments on dyes with TiO_2_. The results show that the prepared TiO_2_ has good photocatalytic activity, which also shows that TiO_2_ has a good effect on the degradation of organic dyes. However, TiO_2_ has the disadvantages of low light energy utilization and low quantum yield, which hinders its large‐scale use. To solve these problems and give full play to the advantages of TiO_2_, researchers have carried out a lot of improvement research.^[11]^ Studies have shown that loading other materials on the surface of TiO_2_ can effectively improve the photocatalytic effect and is more conducive to the degradation of dyes.^[12]^ Therefore, the research on TiO_2_ composites has always been a hot topic.

Covalent organic frameworks (COFs) are a class of crystalline porous materials formed by connecting organic units through chemical covalent bonds. They have the advantages of clear composition, high specific surface area, low density, and good thermal stability.^[13]^ In recent years, COFs have shown great application potential in the field of photocatalysis.^[14]^ He et al^[15]^ prepared COF materials and carried out photocatalytic degradation experiments on dyes such as methyl orange and methylene blue with COFs. The results showed that the material had good photocatalytic degradation performance. The COF material synthesized by Bhadra et al,^[16]^ due to its stability, can maintain good photocatalytic activity in multiple photocatalytic reaction experiments. It can be seen that COF materials have great development potential in the field of photocatalysis. In addition to exerting its advantages, COF materials can also be combined with other photocatalytic materials to increase the effect of photocatalytic degradation. He et al^[17]^ Designed a COF hybrid material to remove Cr (VI), and the removal rate reached 99 % within 20 minutes. The removal rate is improved by multi‐element doping. It can be seen that the photocatalytic degradation performance of the composite material is more advantageous than that of the single material.

In this paper, the advantages of COFs and TiO_2_ were combined to prepare a TiO_2_/COF composite. Then, they are characterized by TEM, XRD, FTIR, and other techniques. Under the irradiation of ultraviolet light, a photocatalytic degradation experiment was carried out using malachite green dye as a simulated dye wastewater, and the first‐order kinetics was investigated. The results show that TiO_2_/COF materials can exert great potential in photocatalytic degradation of dyes, and also have potential utilization value in dye wastewater treatment.

## Results and Discussion

### Scanning Electron Microscopy (SEM) and Transmission Electron Microscope (TEM)

**Figure 1:**
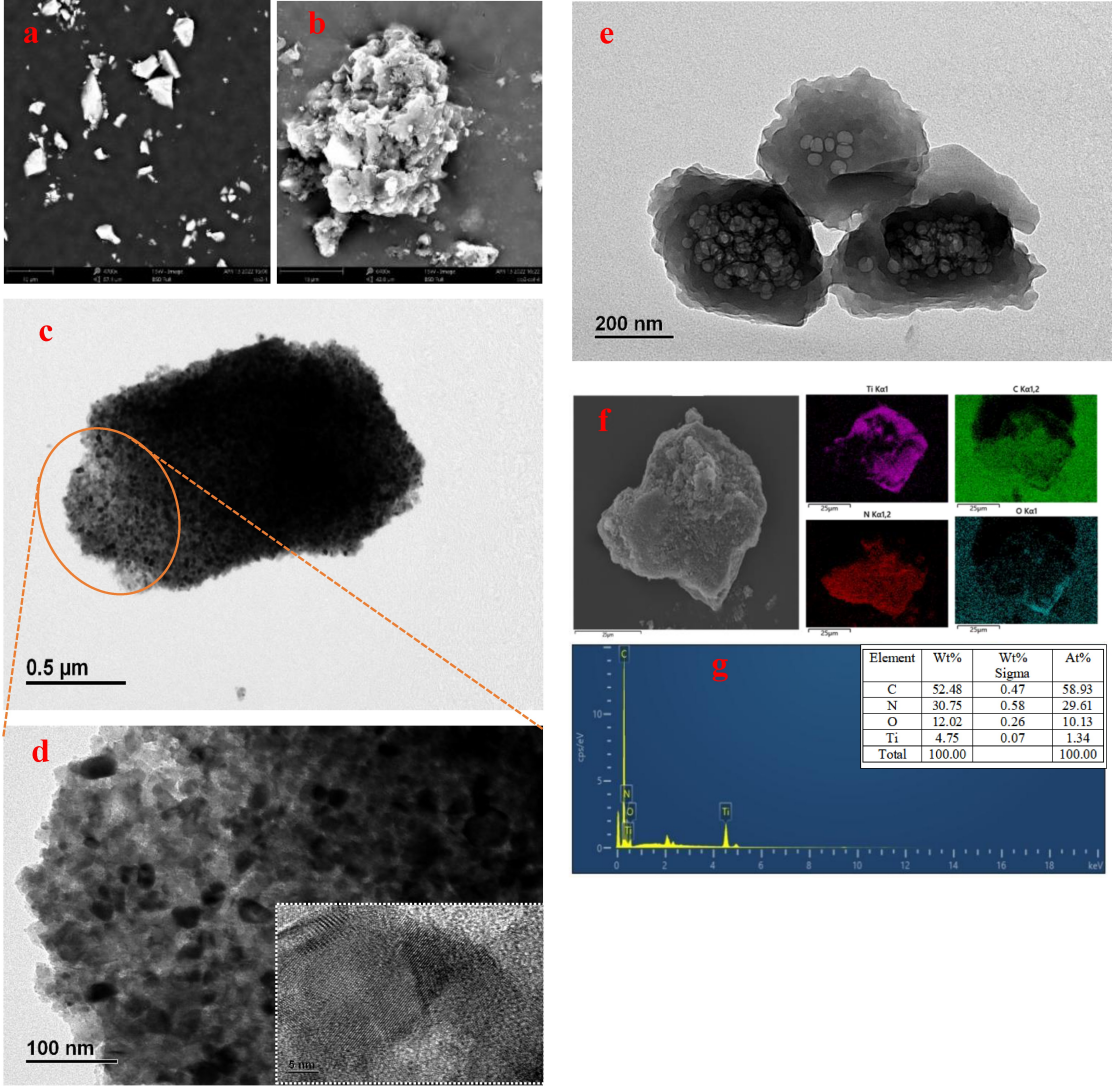
SEM images of TiO_2_ (a) and TiO_2_/COF (b); TEM images of TiO_2_ (c–d) and TiO_2_/COF (e); Mapping EDS of TiO_2_/COF (f–g).

The morphologies of TiO_2_ and TiO_2_/COF were observed by SEM and TEM. From the SEM image of TiO_2_ (Figure 1a), it can be seen that the shape of TiO_2_ prepared by the sol‐gel method is mostly block‐like, with irregular shape and smooth surface. To further determine the morphology of TiO_2_, TEM was used to test TiO_2_. It can be seen from Figure 1c–d that TiO_2_ is elliptical, and the lattice stripes of TiO_2_ can be seen from the inset of Figure 1d. From the SEM image of TiO_2_/COF (Figure 1b), it can be seen that the surface of the material is rough and has certain pores, but the pore size distribution is not uniform enough. To further determine the morphology of TiO_2_/COF, TEM was used to test TiO_2_/COF. As can be seen from Figure 1e, the COF surface is rough, and elliptical TiO_2_/COFs are loaded on the COF surface. The changes in surface morphology of TiO_2_ and TiO_2_/COF indicate the successful preparation of TiO_2_/COF. It can be seen from the mapping EDS image of TiO_2_/COF (Figure 1f) that TiO_2_/COF is mainly composed of C, N, O, and Ti. The map sum spectrum (Figure 1g) of TiO_2_/COF shows that the weight percentages of C, N, O, and Ti elements in TiO_2_/COF are 52.48 %, 30.75 %, 12.02 %, and 4.75 % respectively. It can be seen that the content of the Ti element is relatively small. This result also confirms that TiO_2_ and COF are successfully combined into a complex.

### Fourier Transform Infrared Spectra (FTIR) and Laser Raman Scattering Spectra (RS)

**Figure 2:**
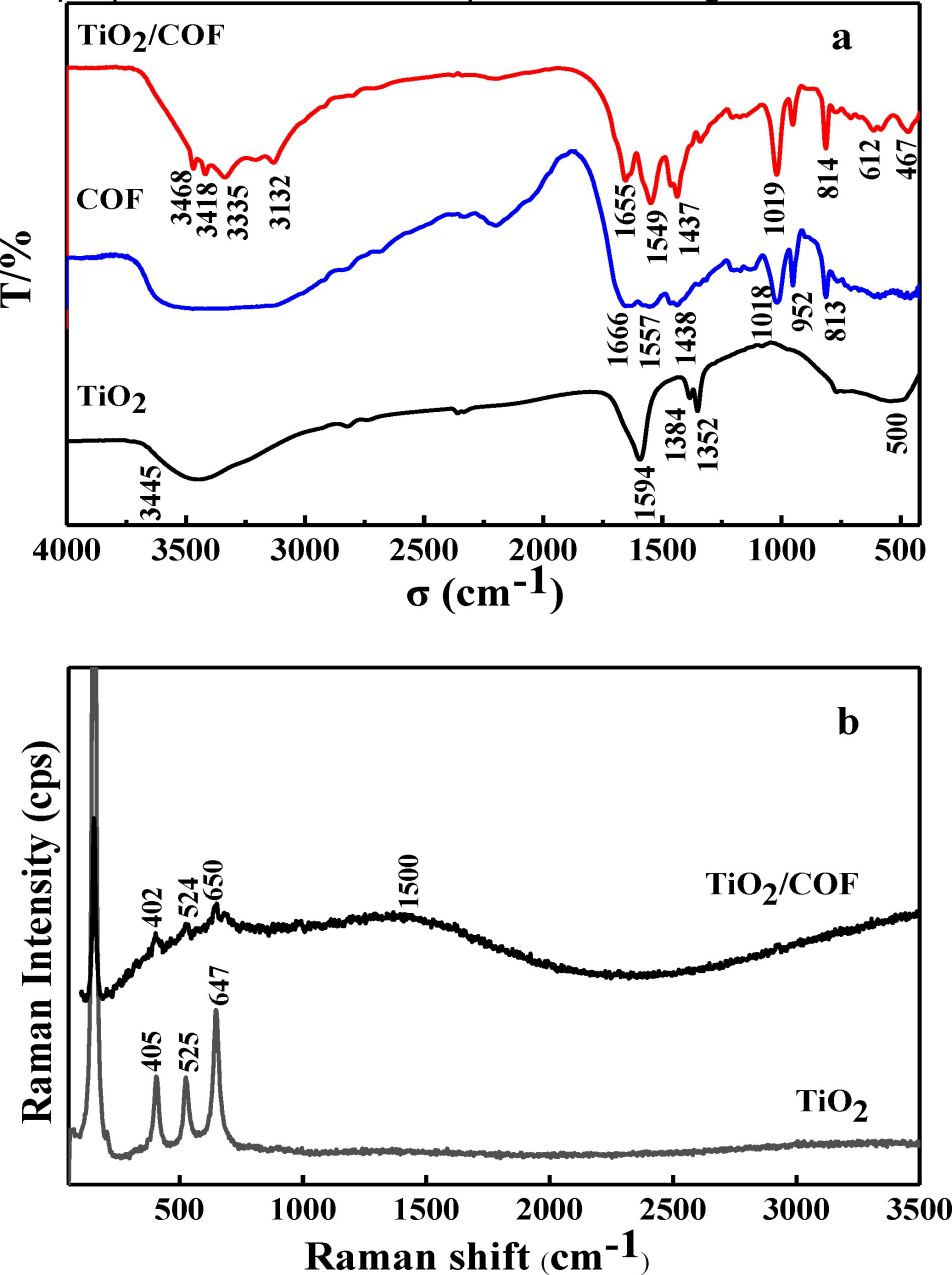
FTIR spectra (a) and RS spectra (b).

The chemical composition of TiO_2_ and TiO_2_/COF was analyzed by FTIR. As shown in Figure 2a, TiO_2_ has an absorption peak near 500 cm^−1^, which is the characteristic absorption peak of TiO_2_.^[18]^ It can be seen from the FTIR of COF (Figure 2a) that COF has an absorption peak at 1018 cm^−1^, which is attributed to the C−N stretching vibration peak. The peak at 1666 cm^−1^ is attributed to the stretching vibration peak of C=O. The absorption peaks at 1557 and 1438 cm^−1^ are attributed to the C=N stretching vibration peak. These results indicate that the COF structure may contain a triazine ring. It can be seen from the FTIR of TiO_2_/COF (Figure 2a) that TiO_2_/COF has absorption peaks at 467, 1019, 1549, 1437, and 1655 cm^−1^ respectively. These absorption peaks are the characteristic absorption peaks of TiO_2_ and COF respectively. The appearance of these absorption peaks indicates that TiO_2_/COF has been successfully synthesized. The laser Raman spectrum is shown in Figure 2b. Both TiO_2_ and TiO_2_/COF materials have Raman scattering peaks of TiO_2_ around 500 cm^−1^. At the same time, a new steamed bread peak appeared around 1500 cm^−1^ in TiO_2_/COF, and the Raman scattering peak was C peak. The reason for the appearance of the steamed bread peak may be due to the interference of fluorescence, which indicates that the prepared TiO_2_/COF complex has strong fluorescence.

### X‐ray Powder Diffraction (XRD) and Thermogravimetric Analysis (TG)

**Figure 3:**
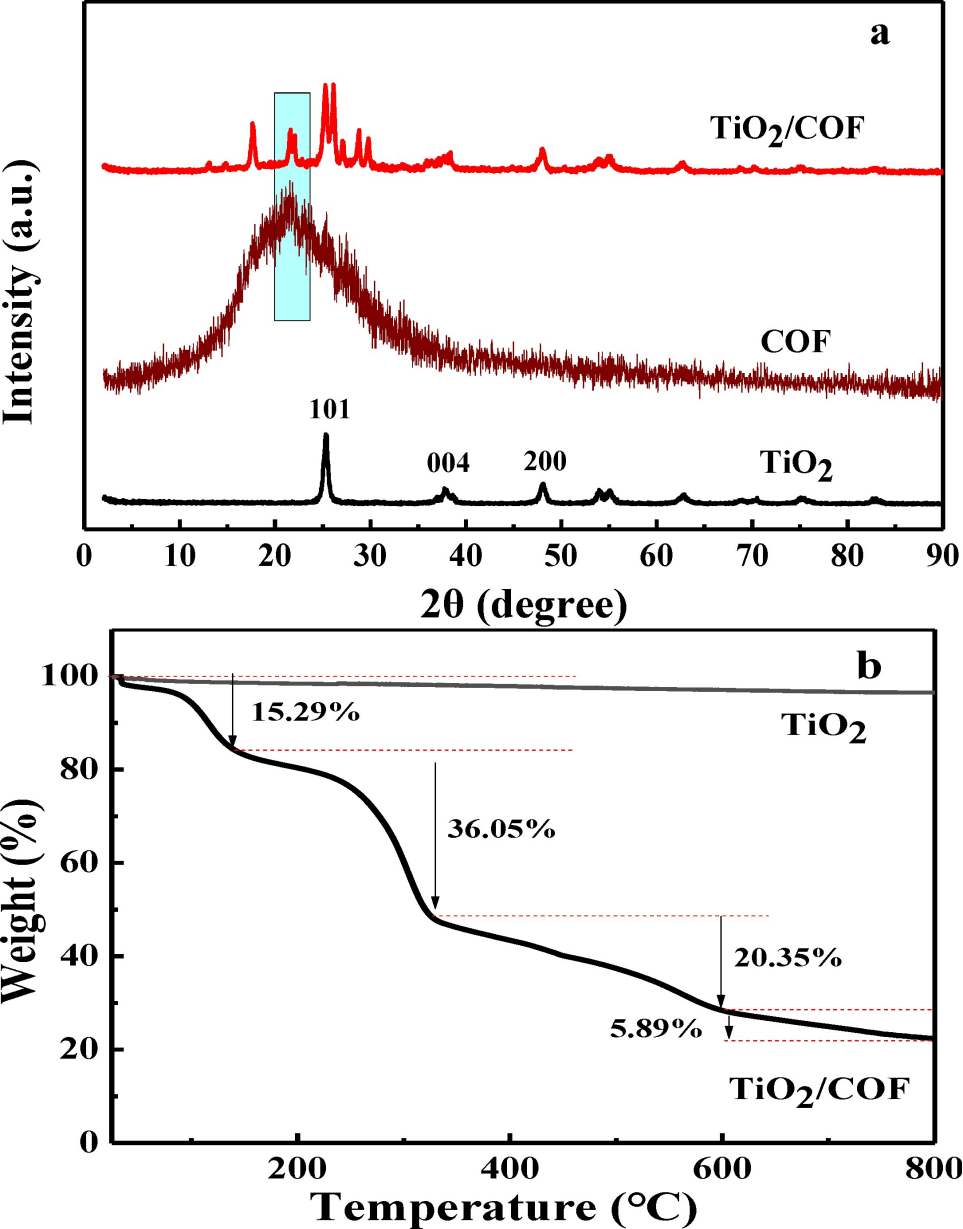
XRD spectra (a) and TG curves (b) of TiO_2_ and TiO_2_/COF.

The crystallinity of the material was determined by XRD. As shown in Figure 3a, TiO_2_ has sharp diffraction peaks at 25.36°, 37.86°, and 48.16°, corresponding to the (101), (004), and (200) crystal planes, respectively. Compared with the XRD pattern of standard TiO_2_ (reference code: 21–1272), it can be seen that the TiO_2_ prepared by the sol‐gel method is anatase type. It can be seen from the XRD diffraction peak of COF that COF has a broad peak at around 22°, and its structure is mainly amorphous with low crystallinity (Figure 3a).^[13]^ It can be seen from the diffraction peaks of TiO_2_/COF that the characteristic peaks of TiO_2_ and COF appear in the TiO_2_/COF composite, which indicates that the crystal structures of TiO_2_ and COF remain intact in the composite. These results indicate that TiO_2_/COF has been successfully prepared.

The thermal stability of TiO_2_ and TiO_2_/COF was investigated by the TG technique, and the results are shown in Figure 3b. It can be seen from the results that the TG curve of TiO_2_ has no obvious weight loss below 800 °C, which indicates that the material exhibits good thermal stability. From the TG curve of TiO_2_/COF, it can be seen that when the temperature is lower than 141 °C, the weight loss of the material is 15.29 %, which is caused by the residual water physically adsorbed on the surface of the sample. When the temperature was between 141 °C and 334 °C, the material weight loss was 36.05 %, which is due to the decomposition of the organic solvent dimethyl sulfoxide in the system. When the temperature is between 334 °C and 601 °C, the material weight loss is 20.35 %, which may be due to the cleavage of the C=N bond in the COF. When the temperature is higher than 601 °C, the weight loss of the material is 5.89 %, which may be due to the disappearance of CO_2_, N_2,_ and other gases generated by C and N molecules. At 800 °C, the remaining 22.42 % is TiO_2_. The results show that TiO_2_/COF has good thermal stability in a certain temperature range.

### Ultraviolet‐Visible Diffuse Reflectance Spectra (UV‐Vis‐DRS)

**Figure 4:**
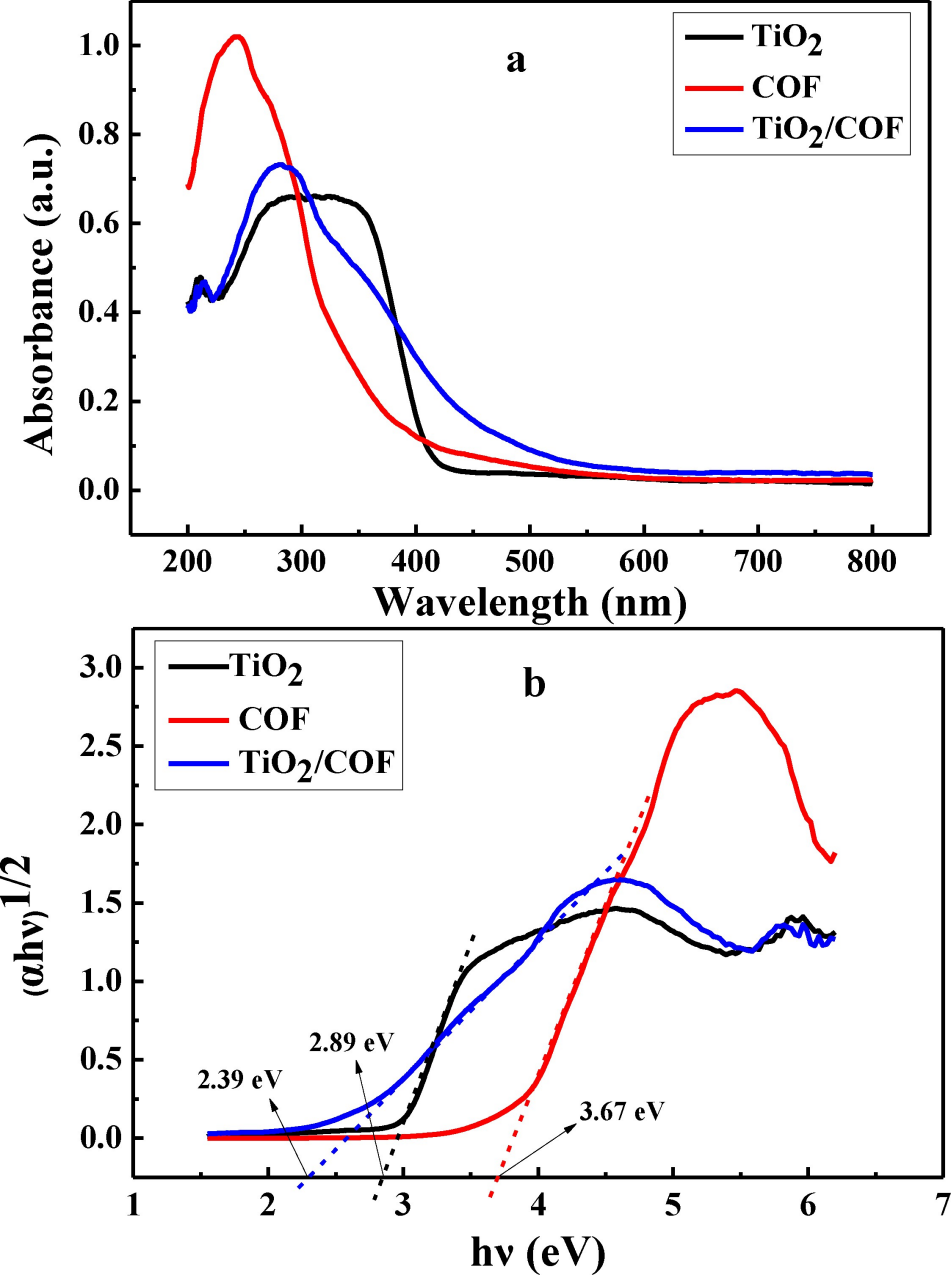
UV‐Vis diffuse reflectance spectra (a) and (*αhν*)^1/2^‐*hν* curves (b).

UV‐Vis diffuse reflectance spectra were used to evaluate the light absorption properties of TiO_2_, COF, and TiO_2_/COF materials, and the results are shown in Figure 4a. It can be seen from the results that the absorption boundaries of TiO_2_ and COF are around 420 nm and 390 nm respectively, while the absorption boundary of the TiO_2_/COF composite is around 477 nm. Moreover, when the wavelength is 200~300 nm, the absorbance of TiO_2_/COF composite is higher than TiO_2_, but weaker than COF, and produces a slight red shift. Therefore, the combination of TiO_2_ and COF can effectively improve the light utilization rate and enhance the degradation effect of dyes.

The optical bandgap energy is the energy required to transfer electrons from the valence band to the conduction band in a semiconductor. The energy is calculated from the UV‐Vis diffuse reflectance results and the Tauc equation:^[19]^

(1)

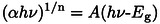




where α is the absorption coefficient, h is Planck's constant, n is the photon frequency, and A is the proportionality constant. There are various values of n in the Tauc equation, and the value of n in this paper is 2.^[20]^ The band gap energy (Eg) of the nanoparticle is obtained by plotting (*αhν*)^1/2^ versus energy (Figure 4b). As can be seen from Figure 4b, the band gap energies of COF, TiO_2_, and TiO_2_/COF are 3.67, 2.89, and 2.39 eV, respectively. The results will be used for the subsequent analysis of the photocatalytic degradation mechanism.

### X‐ray Photoelectron Spectroscopy (XPS)

**Figure 5:**
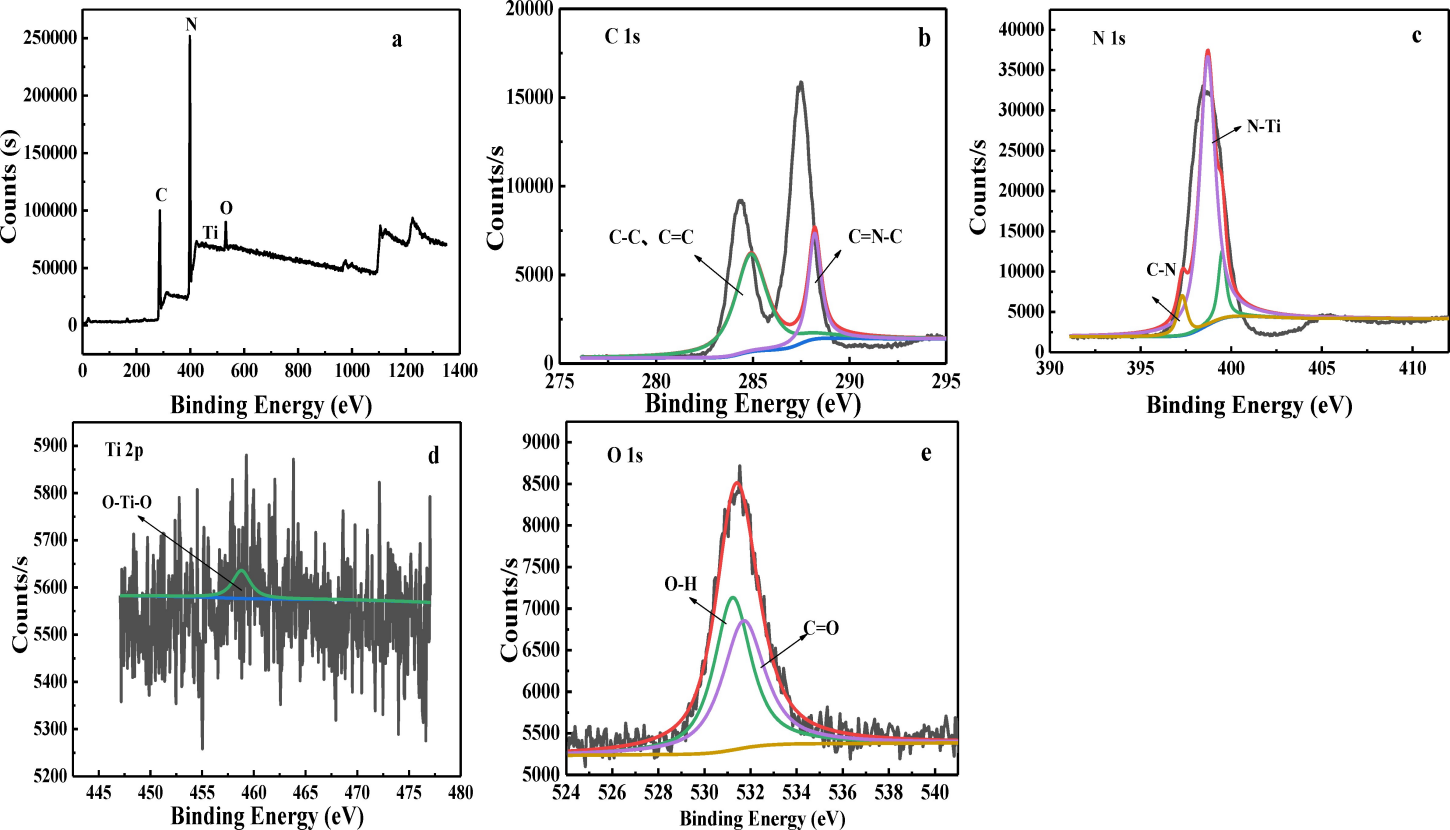
XPS spectra of TiO_2_/COF composite. (a) survey, (b) C 1s, (c) N 1s, (d) Ti 2p, and (e) O 1s.

The elemental composition and existing state of TiO_2_/COF were analyzed by XPS, and the results are shown in Figure 5. It can be seen from the analysis in Figure 5a that there are C 1s peak (285.0 eV), N 1s peak (398.4 eV), Ti 2p peak (460.0 eV), and O 1s peak (531.8 eV) in the survey spectrum. The results show that TiO_2_/COF contains C, N, Ti, and O four elements. Figure 5b is the C 1s spectrum of TiO_2_/COF, the peak with a binding energy of 284.9 eV corresponds to C−C and C=C bonds, and the peak with a binding energy of 288.2 eV corresponds to C=N−C bong.^[21]^ Figure 5c is the N 1s spectrum of TiO_2_/COF, the main peak corresponds to the N−Ti bond with a binding energy of 398.7 eV, and the peak with a binding energy of 399.5 eV corresponds to the C−N bond. Figure 5d is the Ti 2p3/2 spectrum of TiO_2_/COF with a binding energy of 458.8 eV. Figure 5e is the O 1s spectrum of TiO_2_/COF, the main peak corresponds to C=O, and the binding energy is 531.7 eV. From the XPS results, it can be seen that TiO_2_ has been successfully loaded onto the COF.

### Pore diameter and pore volume analysis

**Figure 6:**
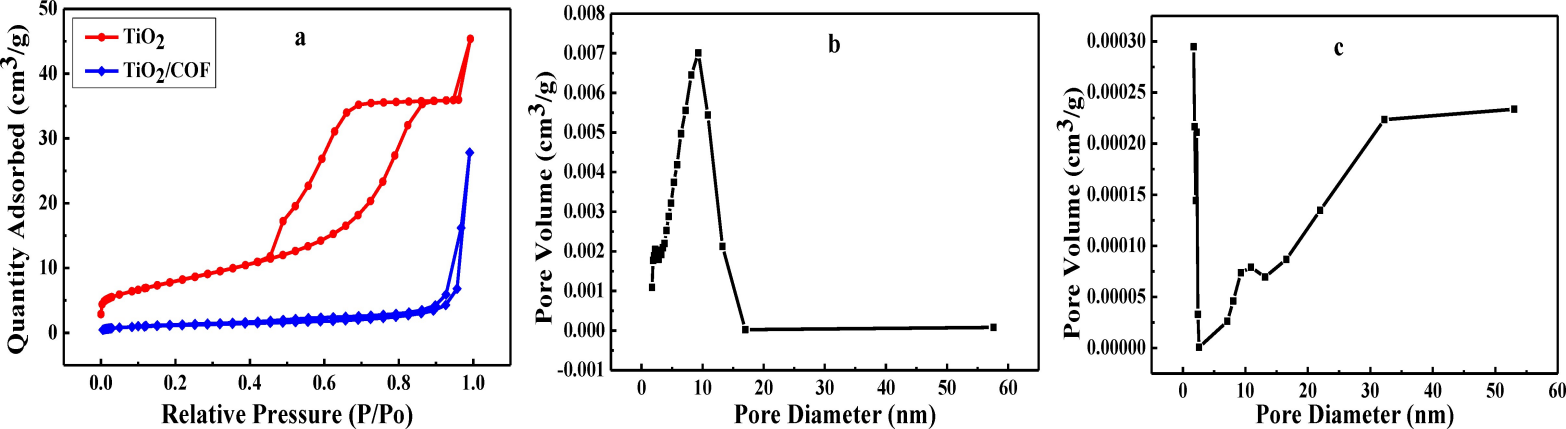
Nitrogen adsorption‐desorption isotherm curve (a), pore size distribution curve of TiO_2_ (b), and pore size distribution curve of TiO_2_/COF (c).

The pore diameter and pore volume of TiO_2_ and TiO_2_/COF were analyzed, and the results are shown in Figure 6. As can be seen from Figure 6a, the N_2_ adsorption‐desorption isotherms of TiO_2_ and TiO_2_/COF are both type IV, and have hysteresis in a relatively high range of pressure. This result shows that both TiO_2_ and TiO_2_/COF have mesoporous structures. The BET surface areas of TiO_2_ and TiO_2_/COF are 28.7873 m^2^/g and 5.3007 m^2^/g respectively, and the pore volumes are 0.070320 cm^3^/g and 0.060095 cm^3^/g respectively. After the TiO_2_ surface is loaded with COF, its surface area and pore volume decrease, which may be due to some pores being blocked. From the TEM image of TiO_2_/COF (Figure 1e), it can also be seen that COF wraps TiO_2_, which may be the reason why the TiO_2_/COF pore channels are restricted and the surface area decreases. Figure 6b–c are the pore size distribution diagrams of TiO_2_ and TiO_2_/COF respectively. It can be seen from the results that the average pore diameter of TiO_2_ is 9.7710 nm, and the average pore diameter of TiO_2_/COF is 41.4675 nm. This result shows that after TiO_2_ is loaded with COF, the pore size of the composite material becomes larger, which also indicates that TiO_2_/COF was successfully prepared.

### Study on Photocatalytic Degradation Performance

**Figure 7:**
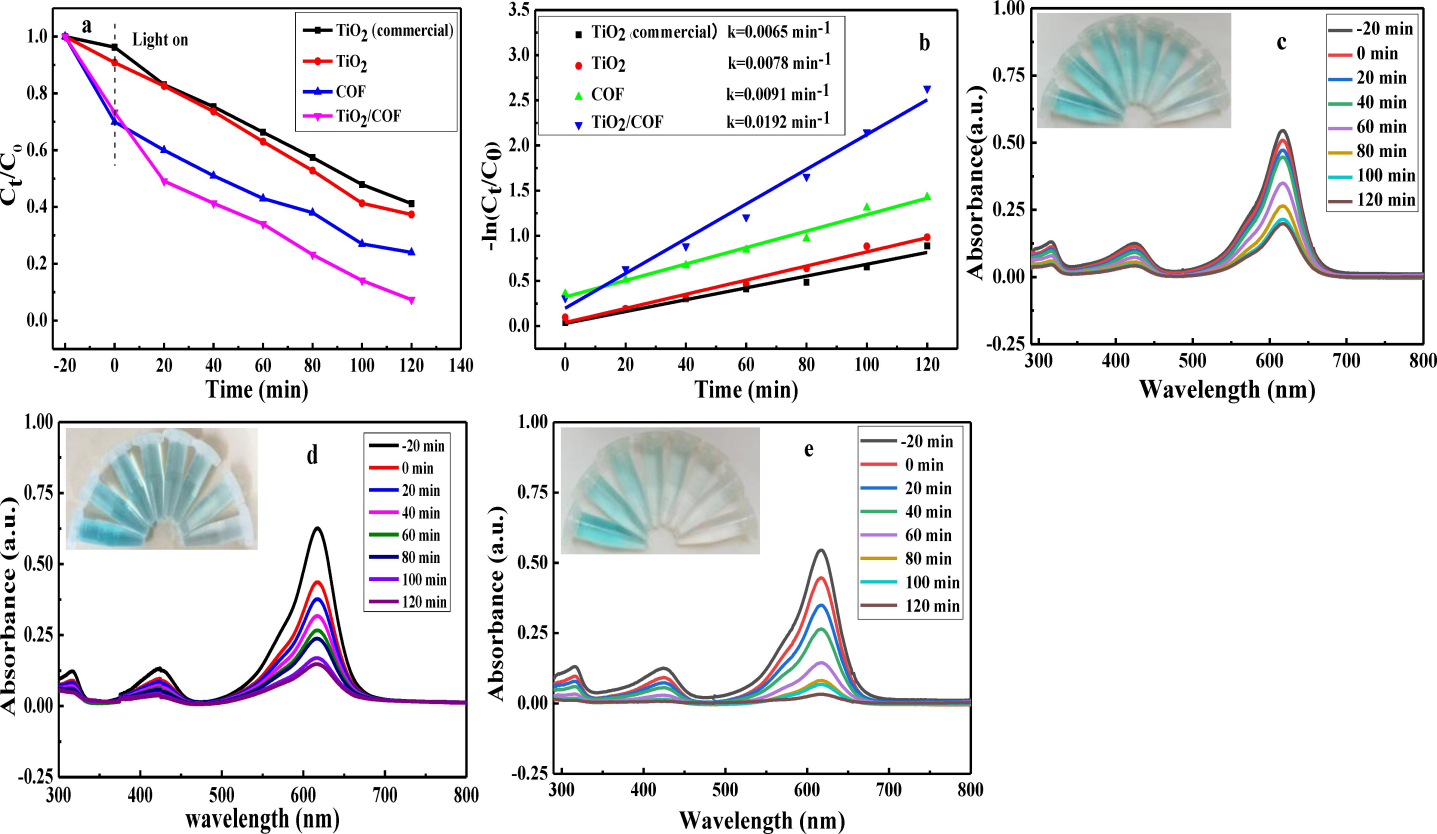
Photocatalytic degradation effect of the system (a), photocatalytic degradation kinetic data (b), and UV‐visible absorption spectra of photocatalytic degradation of malachite green by TiO_2_ (c), COF (d) and TiO_2_/COF (e).

To evaluate the photocatalytic properties of the materials, TiO_2_ (commercial), TiO_2_, COF, and TiO_2_/COF were used as photocatalysts to investigate their photocatalytic degradation effects on malachite green. Under the optimal conditions (the catalyst dosage was 1.67 g/L, and the malachite green concentration was 10 ppm), the photocatalytic degradation experiment was carried out after the dark reaction reached the adsorption equilibrium for 20 min. It can be seen from Figure 7a that in the dark reaction stage, the curves all decrease to a certain extent, which is the reason for the adsorption. Among them, COF has the highest adsorption efficiency. In the photocatalytic degradation experiment, the degradation rates of TiO_2_ (commercial), TiO_2_, COF, and TiO_2_/COF were 55.80 %, 69.77 %, 76.45 %, and 93.64 % respectively. Among them, the TiO_2_/COF composite has the highest photocatalytic degradation rate, which indicates that the TiO_2_/COF composite has the best photocatalytic activity. The UV‐visible absorption spectra of TiO_2_, COF, and TiO_2_/COF photocatalytic degradation of malachite green are shown in Figure 7c–e. It can be seen from the results that after 120 min of UV irradiation, TiO_2_, COF, and TiO_2_/COF can gradually degrade malachite green from green to light green or colorless, and the UV‐visible absorption signals of the solution gradually weaken. Among them, TiO_2_/COF has the best photocatalytic degradation effect on malachite green. This phenomenon also shows that TiO_2_/COF has the best photocatalytic activity.

In addition, the photocatalytic behavior of TiO_2_/COF composites for malachite green conforms to the first‐order kinetic equation, and the results are shown in Figure 7b. The linear relationship obtained by plotting ‐ln(C_t_/C_0_) against t is –ln(C_t_/C_0_)=0.0192 t+0.1988, R^2^=0.9842, and the reaction rate constant is 0.0192 min^−1^. Among them, R^2^ is greater than 0.95, reflecting that –ln (C_t_/C_0_) – t has a good linear correlation.^[22]^ The composite of TiO_2_ and COF improves the photocatalytic activity of TiO_2_, has the characteristics of first‐order kinetics, and is more conducive to the degradation of dye wastewater.

The photocatalytic degradation activity of TiO_2_/COF on malachite green was studied using a low‐power UV lamp at room temperature. Compared with other reported methods for photocatalytic degradation of malachite green (Table 1), this method is simple, cost‐effective, and has high photocatalytic activity. It is expected to be used in the treatment of dye wastewater in the future.

**Table 1:** Comparison of performance of photocatalytic degradation of malachite green.

Catalyst	Light power	Irradiation time	degradation rate	rate constant k	characteristics	references
Ag_2_O	solar	180 min	83 %	–	environmentally friendly, cost‐effective	[23]
NiO/zeolite	LED light (460 nm)	240 min	96 %	–	high degradation efficiency	[24]
WO_3_/Eu_2_O_3_	visible light (425 nm)	80 min	40 %	0.0061 min^−1^	clean and environmentally friendly	[25]
Cr‐TiO_2_@Fe_3_O4	solar	300 min	60 %	0.0099 min^−1^	low cost	[26]
rGO/ZnO	solar	140 min	–	0.0247 min^−1^	good catalytic activity	[27]
CZnO	mercury lamp (254 nm)	60 min	90 %	0.0090 min^−1^	green synthesis	[28]
rGO‐CoSe	solar	200 min	–	0.0105 min^−1^	high degradation efficiency	[29]
carbon dots	mercury lamp (254 nm)	80 min	98.25 %	0.0013 min^−1^	green synthesis, but with a small K value	[30]
(CeO2)cs/(CeO2)gs	UV light	180 min	97.5 %/71 %	–	green synthesis, but the catalyst dosage is large	[31]
TiO_2_/COF	UV light	120 min	93.64 %	0.0192 min^−1^	simple, cost‐effective, high degradation rate	this method

### Recycling Utilization of Photocatalysts

**Figure 8:**
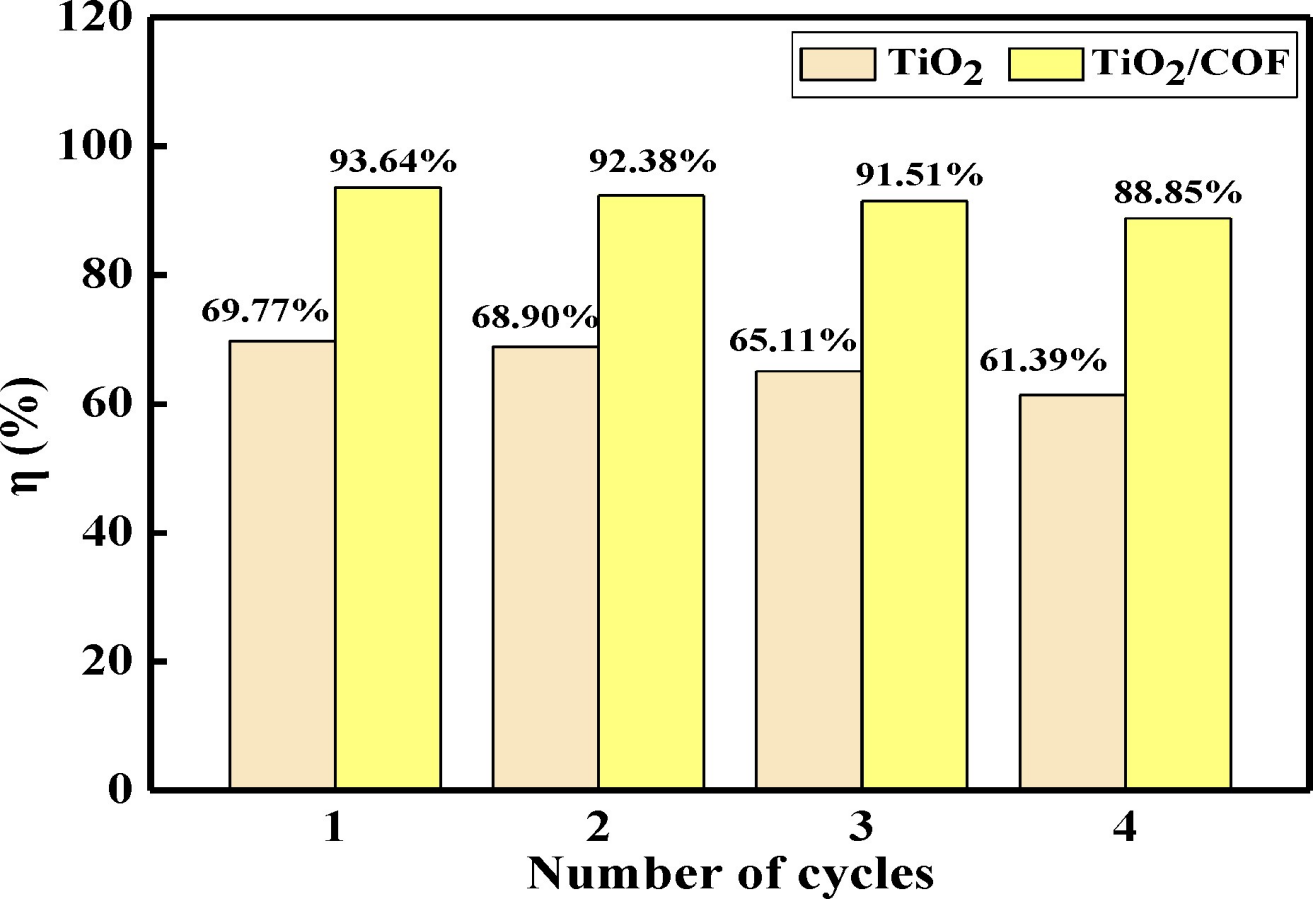
Recycling utilization of TiO_2_ and TiO_2_/COF.

The stability of the material was checked by examining the recycling rate of TiO_2_ and TiO_2_/COF. Under optimal conditions, photocatalytic degradation experiments were carried out. After every 120 min of illumination, the photocatalysts were recovered by centrifugation. The catalysts were first washed with anhydrous ethanol and then centrifuged, and the processes were alternately performed three times. Finally, they were dried at 60 °C and used for the next photocatalytic experiment. The recycling of the photocatalyst is shown in Figure 8. It can be seen from the results that the degradation rates of TiO_2_ and TiO_2_/COF to malachite green only decrease slightly with the increase in the number of cycles. After three cycles, the degradation rates of TiO_2_ and TiO_2_/COF composites are 61.39 % and 88.85 %, respectively. The results show that TiO_2_ and TiO_2_/COF composites have good stability and reusability.

### TiO_2_/COF Photocatalytic Degradation Mechanism

**Figure 9:**
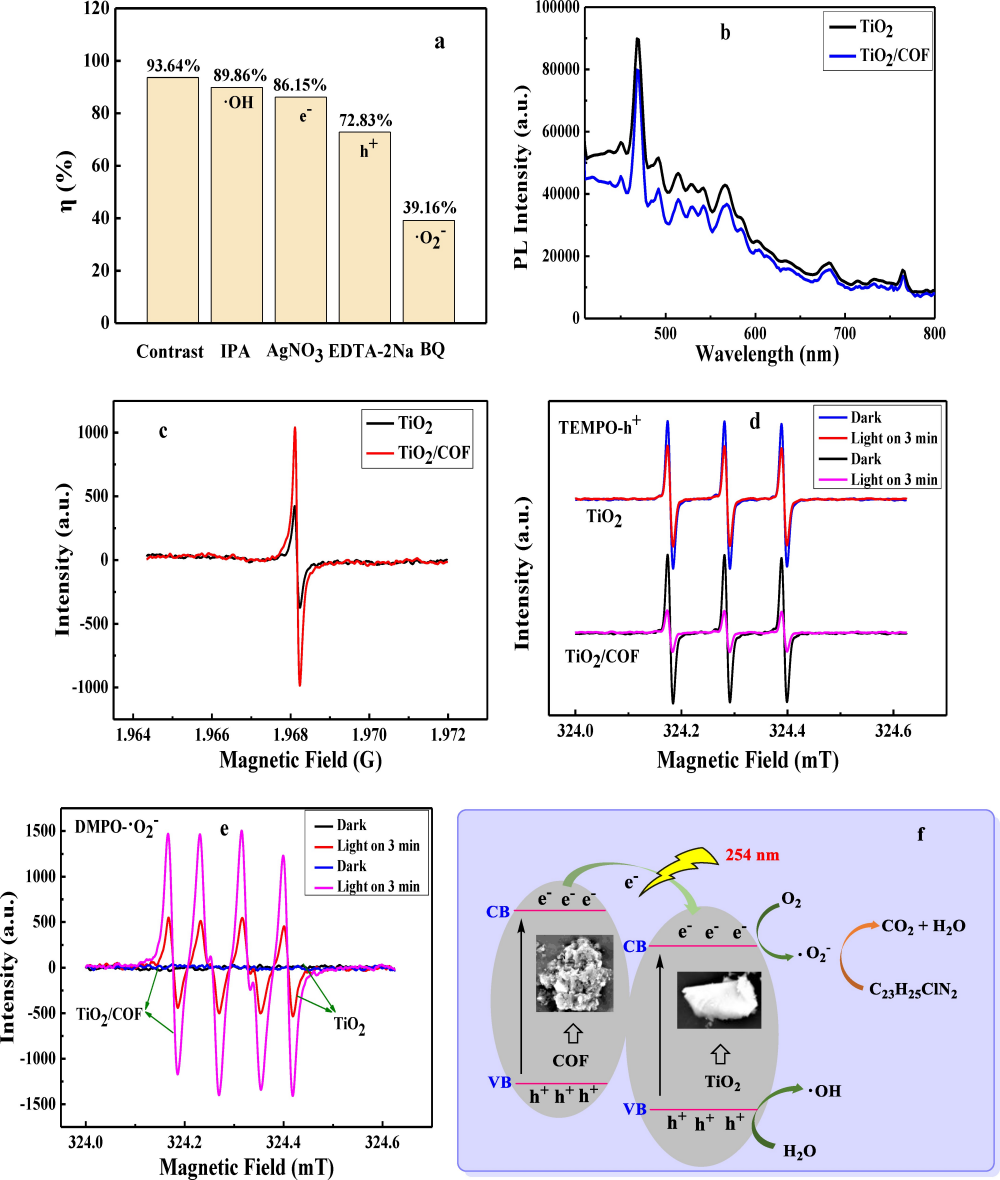
Free radical trapping effect of TiO_2_/COF (a), Steady‐state PL spectra (b), EPR spectra of TiO_2_ and TiO_2_/COF (c), EPR spectra of TiO_2_ and TiO_2_/COF capturing h^+^ (d); EPR spectra of TiO_2_ and TiO_2_/COF capturing ⋅ O_2_^−^ (e), and photocatalytic degradation mechanism of TiO_2_/COF (f).

To study the main active substances formed during photocatalytic degradation, the free radical capture experiments of TiO_2_/COF composites were carried out by adding scavengers. IPA, BQ, AgNO_3_, and EDTA‐2Na were used as scavengers for ⋅ OH, ⋅ O_2_^−^, e^−^, and h^+^ respectively. In the presence of BQ and EDTA‐2Na, the degradation rates of malachite green were significantly inhibited (Figure 9a), and the degradation rates were 72.83 % and 39.16 % respectively, which indicate that ⋅ O_2_^−^ and h^+^ are the main active substances that control the degradation process of malachite green. Furthermore, slight inhibition can be observed when IPA and AgNO_3_ are added, and the degradation rates of malachite green are 89.86 % and 86.15 % respectively. The results indicate that ⋅ OH and e^−^ have little influence on the photocatalytic degradation of malachite green by TiO_2_/COF.

Photoluminescence spectroscopy (PL) was used to characterize the migration and recombination process of photogenerated electron‐hole pairs in photocatalysts. As shown in Figure 9b, under the condition of an excitation wavelength of 275 nm, TiO_2_ has a strong fluorescence emission intensity, and after loading COF, the fluorescence is quenched. This shows that the introduction of COF into TiO_2_ can effectively increase the carrier transmission rate and thereby inhibit photogenerated electron‐hole recombination, and the photocatalytic activity of the TiO_2_/COF composite is improved.

Electron paramagnetic resonance (EPR) technology was used to study the charge transfer process. Both TiO_2_ and TiO_2_/COF exhibit a single Lorentz line (g=1.968), which is considered to be the lone pair of electrons of the material (Figure 9c). Compared with TiO_2_, the peak intensity of TiO_2_/COF increases significantly, which indicates that the concentration of lone pair electrons is higher and the charge transfer is improved. This result is consistent with the photocatalytic ability of TiO_2_ and TiO_2_/COF towards malachite green. The photocatalyst was dispersed in 2,2,6,6‐tetramethylpiperidine‐1‐oxide (TEMPO) acetonitrile solvent, and the change of h^+^ in the photocatalyst was observed. The results are shown in Figure 9d. There are three strong signals in the figure corresponding to the h^+^ characteristic peak. As the irradiation time increases, h^+^ consumes more capture agents, the capture agent signal becomes weaker, and the peak also becomes weaker. Then, the photocatalyst was dispersed in 5,5‐dimethylpyrroline N‐oxide (DMPO) methanol solvent, and the change of ⋅ O_2_^−^ in the photocatalyst was observed. The results are shown in Figure 9e. There are four strong signals in the figure corresponding to the ⋅ O_2_^−^ characteristic peaks. Compared with TiO_2_, the signal intensity of TiO_2_/COF is significantly enhanced, which indicates that the ⋅ O_2_^−^ concentration increases. The higher ⋅ O_2_^−^ concentration in TiO_2_/COF indicates that the efficient photogenerated charge separation efficiency is beneficial to improving the efficiency of TiO_2_/COF photocatalytic reduction of malachite green.^[32]^ These detection results are consistent with the above‐mentioned free radical capture experimental results.

Based on the above results, a possible mechanism for the photocatalytic degradation of malachite green by TiO_2_/COF composites is proposed (Figure 9f). Due to its narrow band gap, COFs are easily excited to generate holes (h^+^) and electrons (e^−^) under UV light irradiation. However, due to the wide band gap, it is difficult for TiO_2_ to generate holes and electrons.^[33]^ Since COF has a more negative conduction band (CB) potential than TiO_2_, a large number of photogenerated electrons in the conduction band of COF will migrate to the conduction band of TiO_2_ with slightly lower energy than COF, which is beneficial to hinder the recombination of electron‐hole pairs. The CB of TiO_2_ is more negative than that of O_2_/ ⋅ O_2_^−^, so the electrons on the CB of TiO_2_ can reduce O_2_ to ⋅ O_2_^−^.^[34]^ And ⋅ OH is produced by the combination of h^+^ and H_2_O.^[20]^ When malachite green was placed on the surface of the photocatalyst, due to the chemical compatibility of the two species, they combined with ⋅ O_2_^−^ and ⋅ OH, respectively, and converted into CO_2_ and H_2_O. Their photocatalytic process can be represented by the following equation:

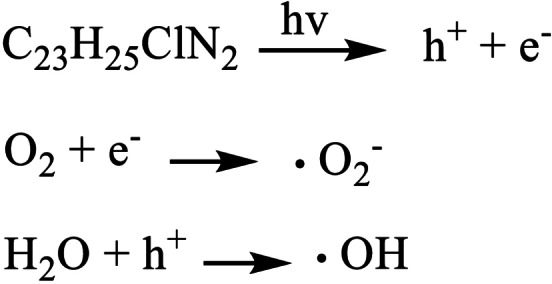




## Conclusions

In this paper, a TiO_2_/COF core‐shell composite was prepared. Malachite green was used as simulated dye wastewater to explore the photocatalytic degradation effect of TiO_2_/COF. The results show that TiO_2_/COF has a significant effect on the photocatalytic degradation of malachite green. Finally, the photocatalytic degradation mechanism of TiO_2_/COF was discussed based on free radical capture experiments, PL, and EPR. The TiO_2_/COF composite has obvious advantages in degrading dye wastewater and has great potential for the treatment of malachite green wastewater.

## Experimental Section

For experimental details, see the Supporting Information section. The authors have cited additional references within the Supporting Information (Ref. [35]).

## Conflict of interests

The authors declare no conflict of interest.

1

## Supporting information

As a service to our authors and readers, this journal provides supporting information supplied by the authors. Such materials are peer reviewed and may be re‐organized for online delivery, but are not copy‐edited or typeset. Technical support issues arising from supporting information (other than missing files) should be addressed to the authors.

Supporting Information

## Data Availability

Data sharing is not applicable to this article as no new data were created or analyzed in this study.
