# Supplementary material for: Photocatalytic Degradation of Malachite Green by Titanium Dioxide/Covalent Organic Framework Composite: Characterization, Performance and Mechanism
Source: ChemistryOpen. 2024 Jan 5;13(6):e202300209. doi: 10.1002/open.202300209 (PMC11164026; doi:10.1002/open.202300209)
Supplement: Supplementary file 1 — Supporting Information [file OPEN-13-e202300209-s001.pdf]

ChemistryOpen

Supporting Information

Photocatalytic Degradation of Malachite Green by Titanium Dioxide/Covalent Organic Framework Composite: Characterization, Performance and Mechanism

Dongmei Yao,* Xiaoting Xie, Xuling Liang, Sufen Lu,* and Hongfang Lai*

Experimental Section

Reagents and Instruments

Melamine, 1,4-phthalaldehyde, and tetrabutyl titanate were purchased from Ron's reagent. Anhydrous ethanol, $AgNO_3$, p-benzoquinone (BQ), disodium ethylenediaminetetraacetate (EDTA-2Na), and isopropanol (IPA) were purchased from Sinopharm Chemical Reagent Co., Ltd. Malachite green (MG) was purchased from Tianjin Guangfu Fine Chemical Research Institute. Glacial acetic acid was purchased from Tianjin Beichen Fangzheng Reagent Factory. The reagents used in the experiments were all analytical grade, and the water used was ultrapure.

8453 UV-Vis Spectrophotometer (Agilent Technologies Co., Ltd., USA), MiniFlex 600 X-ray Powder Diffractometer (Rigaku, Japan), NICOLET 6700 Fourier Transform Infrared Spectrometer (Bruker Spectrometer Co., Ltd., Germany), Scanning Electron Microscope (Phenom Scientific Instruments Co., Ltd., Holland), TG/DTA 6300 Differential Thermogravimetric Analyzer (EXSTAR, Japan), ESCALAB 250Xi X-ray photoelectron spectroscopy (Thermo Fisher Scientific, USA), Tecnai G2 F20 field emission transmission electron microscope (FEI, USA), JES FA200 electron paramagnetic resonance tester (JEOL Ltd., Japan), FLs980 Fluorescence Lifetime and Steady State Spectrometer (Edinburgh Instrument Company, UK), UV-3600 UV-visible spectrophotometer (Shimadzu, Japan), Three-station full-featured multi-purpose adsorption instrument (Micromeritics, USA) were used.

Preparation of TiO_2

In a clean beaker A, sequentially add 10 mL of tetrabutyl titanate and 35 mL of anhydrous ethanol, shake well, and stir at room temperature for 1 h. In another beaker B, add 2 mL of glacial acetic acid, 35 mL of anhydrous ethanol, and 10 mL of H_2O in sequence, shake well, and adjust the pH of the solution to ≤3 with glacial acetic acid. The solutions in the two beakers are mixed, shaken, and stirred in a water bath at 60 °C for 1 h to obtain a milky white gel. [1] After suction filtration and drying, light yellow crystals are obtained. After grinding, it is transferred to a crucible for the high-temperature reaction for 3 h, then cooled and ground to obtain nano-TiO_2.

Preparation of TiO_2/COF

Weigh 0.40 g melamine and 0.10 g 1,4-phthalaldehyde, add 10 mL of dimethyl sulfoxide, dissolve by ultrasonic, then add 10 mL of H_2O and 0.1 g TiO_2, and mix well. Then, the solution is transferred to a crucible, evaporated to dryness at 100 °C, and then transferred to a muffle furnace for heating at 180 °C for 3 h. After cooling to room temperature, the product is washed with H_2O and anhydrous ethanol until neutral. Finally, the TiO_2/COF composites are obtained by vacuum freeze-drying for 48 h.

Photocatalytic Degradation Experiment

Malachite green is used as a simulated dye wastewater to evaluate the photocatalytic degradation activity of TiO_2/COF. The photocatalytic reaction is carried out in a self-made photocatalytic reaction box with an illumination wavelength of 254 nm. Add 50 mg of TiO_2/COF composite (catalyst dosage is 1.67g/L) to a quartz round-bottomed flask containing 30 mL of 10 mg/L (10 ppm) malachite green solution and shake well. After the reaction reaches adsorption equilibrium in the dark for 20 minutes, turn on the UV lamp, take about 2 mL of the supernatant every 20 min, filter, and then transfer it to a quartz cuvette to measure its absorbance with a UV-Vis spectrophotometer.

The relevant calculations for the experiment are as follows:

(1) Degradation rate (η)

$$\eta=\frac{C_0\text{-}C_t}{C_0}\times 100\%=\frac{A_0\text{-}A_t}{A_0}\times 100\%$$

Where C_0 is the initial concentration, C_t is the concentration at time t, A_0 is the initial absorbance, and A_t is the absorbance at time t.

(2) First-order kinetic equation

$$-\ln\left(\frac{C_t}{C_0}\right)=kt$$

Where k is the reaction rate constant, t is the time, C_0 is the initial concentration, and C_t is the concentration at time t.

Photocatalytic Capture Experiment

The photocatalytic degradation mechanism of TiO_2/COF materials is explored by designing free radical capture experiments. Under optimal conditions, isopropanol (IPA), $AgNO_3$, disodium ethylenediaminetetraacetate (EDTA-2Na) and p-benzoquinone (BQ) are used as scavengers to capture hydroxyl radicals (·OH), photogenerated electrons (e^-), photogenerated holes (h^+) and superoxide radicals ($\cdot O_2^-$) in the photocatalytic process, respectively. After adsorption equilibrium, 1 mL of 0.2 mmol/L scavenger (the masses of IPA,

$AgNO_3$, EDTA-2Na, and BQ are 12.0 μg, 33.9 μg, 67.2 μg, and 21.6 μg respectively) was added, shake well, and then the photocatalytic capture experiment was carried out.

[1] L. Chen, K. Zheng, Y. Liu, *Constr. Build Mater.* **2017**, 151, 63-70.
